# The Imaginal Characters of *Neoephemera projecta* Showing Its Plesiomorphic Position and a New Genus Status in the Family (Ephemeroptera: Neoephemeridae)

**DOI:** 10.3390/insects12080723

**Published:** 2021-08-12

**Authors:** Zhenxing Ma, Changfa Zhou

**Affiliations:** College of Life Sciences, Nanjing Normal University, Nanjing 210023, China; 161202021@njnu.edu.cn

**Keywords:** *Pulchephemera* gen. n., new genus, new combination, phylogeny, mayfly, China

## Abstract

**Simple Summary:**

The phylogenetically problematic Neoephemeridae is a small family of the order Ephemeroptera, including four genera reported previously. They are genera *Neoephemera* (in Nearctic region), *Ochernova* (Central Asia), *Leucorhoenanthus* (West Palearctic) and *Potamanthellus* (East Palearctic and Oriental regions), which were geographically isolated until the *Neoephemera projecta* Zhou and Zheng, a species reported in 2001 from Southwestern China, connected them together. In this work, the imaginal stage and biology of *Neoephemera projecta* are first observed and described. Furthermore, a series of autapomorphies and plesiomorphies of it are recognized and discussed. Morphologically and biologically, this species is significantly different from other genera and deserves a new plesiomorphic position in the Family Neoephemeridae. Therefore, a new genus *Pulchephemera* gen. n. is established to reflect its primitive position and intermediate characters of two clades of the family. In addition, some shared characters of this new genus and the family Ephemeridae provide a new perspective or possibility on the phylogeny of Neoephemeridae within the order Ephemeroptera.

**Abstract:**

The newly collected imaginal materials of the species *Neoephemera projecta* Zhou and Zheng, 2001 from Southwestern China, which is linking the other genera of the family Neoephemeridae, are described in detail. Nymphs are also photographed for the first time. The morphology of this species shows some characters of the other genera in Neoephemeridae and several autapomorphies. However, most characters can be seen as plesiomorphies of the family. Specifically, the dorsal-oriented fimbriate gills, projected frons and slim labial palpi in nymphs plus large reddishly pigmented wings, many crossveins, 4-segmented forceps with a relatively long basal segment, fused penes and unforked anal vein show that this species is closer to the taxon Fossoriae rather than to the previously considered Potamanthidae. To reflect its primitive position, a new genus, *Pulchephemera* Zhou gen. n., is established for this species, *Pulchephemera projecta* comb. n. Its eggs and observed biology are also described.

## 1. Introduction

The mayfly family Neoephemeridae (Insecta: Ephemeroptera) includes 13 extant species [1,2,3,4]. Most species have been described from both imaginal and nymphal materials [1,3,5,6]. An exception is the species *Neoephemera projecta* Zhou and Zheng, 2001, the single species of the genus *Neoephemera* McDunnough, 1925 found in China and the Eastern Palearctic region, which was originally reported based on nymphs only [2,7].

Bae and McCafferty [1] reviewed the family Neoephemeridae and differentiated three genera within it: *Neoephemera*, *Ochernova* Bae and McCafferty, 1998 and *Potamanthellus* Lestage, 1931 [8]. In this revision, genus *Leucorhoenanthus* Lestage, 1931 (including one European species *L. maximus* (Joly, 1871)) was regarded as a synonym of *Neoephemera* (including the four Nearctic species at that time) and a new genus *Ochernova* was established for the Central Asian species *Neoephemera tshernovae* Kazlauskas, 1963 [9]. However, the genus classification of Neoephemeridae is still controversial. In the system of Kluge [5], *L. maximus* was still retained as a monospecific taxon Leucorhoenanthus/g1 (= genus *Leucorhoenanthus*) and this system shows the morphological and zoogeographic distribution gap between Nearctic and Palearctic neoephemerid species. It is believed that the imagoes of *N. projecta* will provide new evidence for the theory of generic classification in Neoephemeridae.

Based on the phylogeny of extant Neoephemeridae in Bae and McCafferty [1], the four genera (*sensu* Kluge [5]) of Neoephemeridae can be divided into two distinct groups: (1) genus *Potamanthellus* with vestigial forceps and pigmented wings; (2) *Neoephemera*-like group (including genera *Neoephemera*, *Ochernova* and *Leucorhoenanthus*), with relatively normal forceps and transparent wings. As the single extant species linking the two groups geographically, the phylogenetic position of *N. projecta* within Neoephemeridae is very important for the generic phylogeny of this family. 

Generally, the morphology of the family Neoephemeridae shows an unstable phylogenetic position and enigmatic origin [5,10,11]. Previously, its adults were regarded as possessing potamanthid-like wing venation and genitalia but its nymphs with caenid-like body and gill pattern [12,13]. As the largest species of this family (with large body size and many plesiomorphies), the detailed description of *N. projecta* will help us test the existing hypothesis or propose a new one for the systematic position of Neoephemeridae.

In order to clarify the position of *N. projecta*, we tried several times to collect the adults of it. Fortunately, in April 2021, tens of males, females and mature nymphs were found and caught by the authors, allowing us to improve our understanding of this species, genus and family. After careful examination, a new genus is established here to include this species and to reflect its plesiomorphic position combining characters of *Potamanthellus* and *Neoephemera*. 

## 2. Materials and Methods

The nymphs of *N. projecta* were collected by hand net, and the adult (including live subimagoes and female imagoes) were collected in and near the creek in the afternoon, all male and most female imagoes were reared from subimagoes indoors. All specimens were stored in ethanol (more than 80%). The materials were examined under a stereomicroscope and photographed with the digital camera system connected with the microscope. Some small structure of nymphs (e.g., setae on mouthpart, gill, terga and femora), subimagoes (tarsal segments) and eggs (dissected from a female imago) were photographed by an SEM (Scanning Electron Microscope). Diagnostic characters and terminology were adopted from Bae and McCafferty [1] and Kluge [5,14]. Abbreviation for wing veins: “C”: Costa; “Sc”: Subcosta; “MA”: Medius anterior; “MP”: Medius posterior; “Rs”: Radial sector; “Cu”: Cubitus; “CuA”: Cubitus anterior; “CuP”: Cubitus posterior; “A”: Anal vein.

In addition, considering the unique nymphal characteristics (the mouthparts and the caudal filament) and lack of adult information [4], the Vietnamese species *Potamanthellus unicutibius* Nguyen and Bae, 2004 is not mentioned and discussed in this article (including the genetic diagnosis and phylogeny analysis). It seems that the species is a new genus and more information is needed to resolve the confusion.

Sample sites of all other materials mentioned in this article: 

*N. purpurea* (Traver, 1931) [15]: two nymphs, South Carolina, no other data;

*N. youngi* Berner, 1953 [16]: two male imagoes three female imagoes, FLORIDA, Okaloosa Co., Blackwater River, 9–11 May 1998, WL & J Peters; 10 nymphs, FLORIDA: Walton Co., Gum Cr. on Hwy. 331, 6 mi. NW. of DeFuniak Springs, 24-IV-1967, W. L. & J. Peters;

*P. chinensis* Hsu, 1936 [17]: 20 male imagoes, 20 female imagoes, 30 nymphs, Dishui Lake, Yanqing County, Beijing, 17–23-vi-2018, ZHANG Wei & MA Zhen-Xing. 

All materials mentioned in the research are deposited in the Mayfly Collection, College of Life Sciences, Nanjing Normal University (NNU), China.

## 3. Results

*Pulchephemera* Zhou, gen. n. 

Type species and species included: *Pulchephemera projecta* (Zhou and Zheng, 2001).

**Figure 1:**
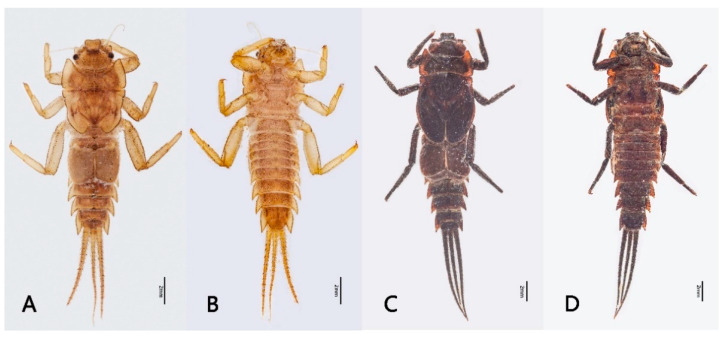
Nymphal habitus of *Pulchephemera projecta*: (**A**) dorsal view (immature nymph); (**B**) ventral view (immature nymph); (**C**) dorsal view (mature nymph); (**D**) ventral view (mature nymph).

**Figure 2:**
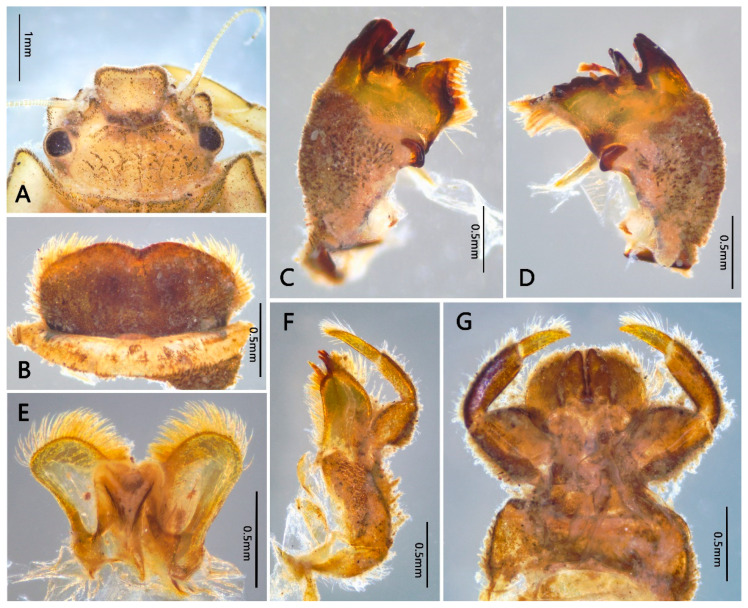
Head and mouthparts of *Pulchephemera projecta*: (**A**) head; (**B**) labrum; (**C**) left mandible; (**D**) right mandible; (**E**) hypopharynx; (**F**) maxillae; (**G**) labium.

**Figure 3:**
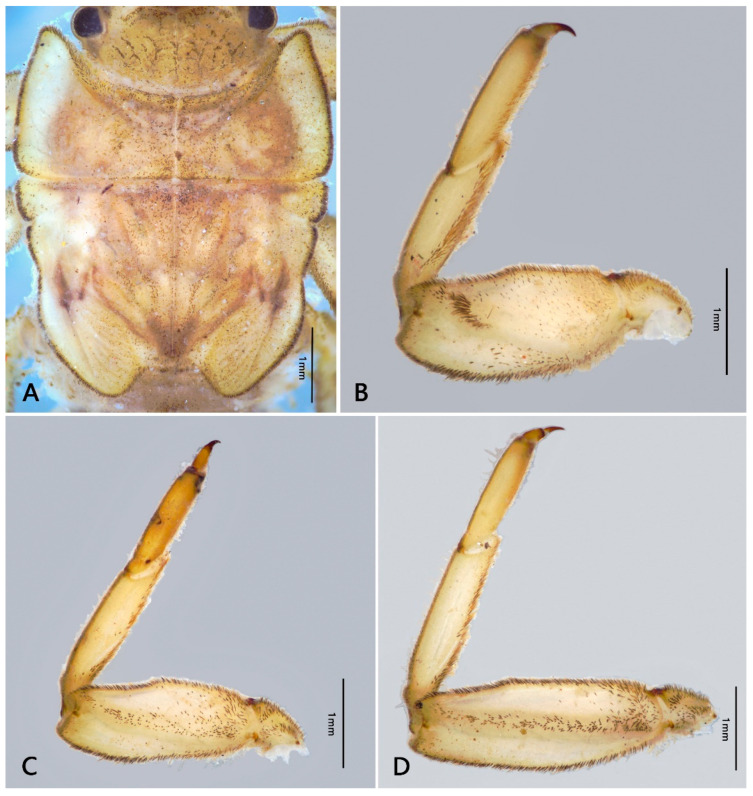
Thoracic structures of *Pulchephemera projecta*: (**A**) thorax; (**B**) foreleg; (**C**) middle leg; (**D**) hind leg.

**Figure 4:**
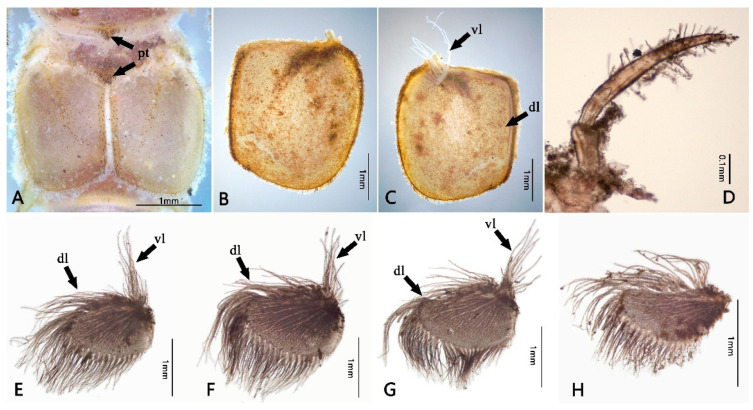
Abdominal structures of *Pulchephemera projecta*: (**A**) abdominal terga I–VI; (**B**) operculate gill II (dorsal view); (**C**) operculate gill II (ventral view); (**D**) gill I; (**E**) gill III; (**F**) gill IV; (**G**) gill V; (**H**) gill VI. Abbreviations: dl, dorsal lamella; vl, ventral lamella; pt, posterior tubercle.

**Figure 5:**
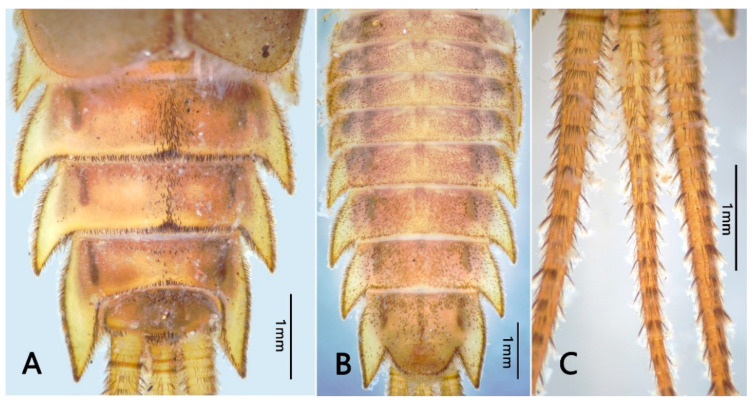
Abdominal structures of *Pulchephemera projecta*: (**A**) abdominal terga VI–X; (**B**) abdominal sterna; (**C**) caudal filaments.

Mature nymph: body longer than 16.0–20.0 mm, generally flat and stout (Figure 1); head with genal and frons projections (Figure 2A), anterior margin of frons concave; leading margin of labrum slightly concave (Figure 2B); maxillary and labial palpi 3-segmented, relatively slender (Figure 2F,G); anterolateral angles of pronotum and mesonotum extended into clear projections; pronotum extended laterally to same level of mesothoracic projections (Figure 3A); two pairs of wingpads present; tibiae of all legs shorter than femora (Figure 3B–D); gills I single, vestigial (Figure 4D); gills II double, operculate, jointed together medially; operculate plate quadrate, with dorsal ridge, ventral lamellae fimbriate (Figure 4B,C); gills III–V with double lamellae, fringed, dorsal lamella sub-oval, much larger than ventral lamella (Figure 4E–G); gills VI single, fimbriate (Figure 4H); posterolateral projections of terga V–IX well developed (Figure 5B); caudal filaments shorter than body, with ring of spine-like setae at articulations only (Figure 5C). 

**Figure 6:**
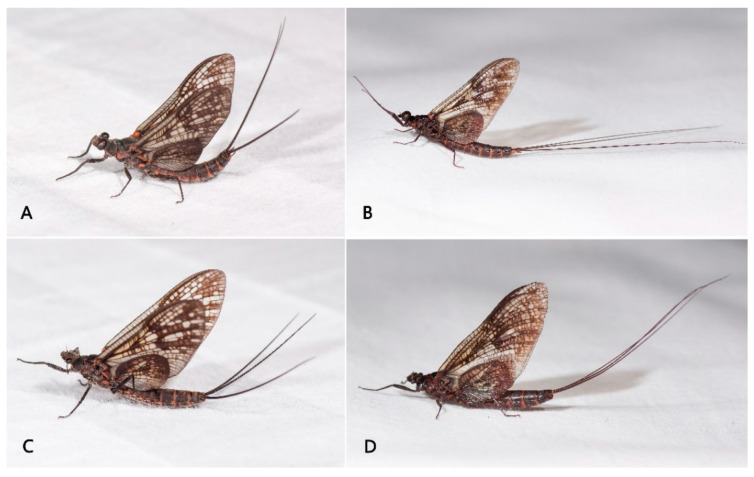
Adult habitus of *Pulchephemera projecta*: (**A**) male subimago; (**B**) male imago; (**C**) female subimago; (**D**) female imago.

**Figure 7:**
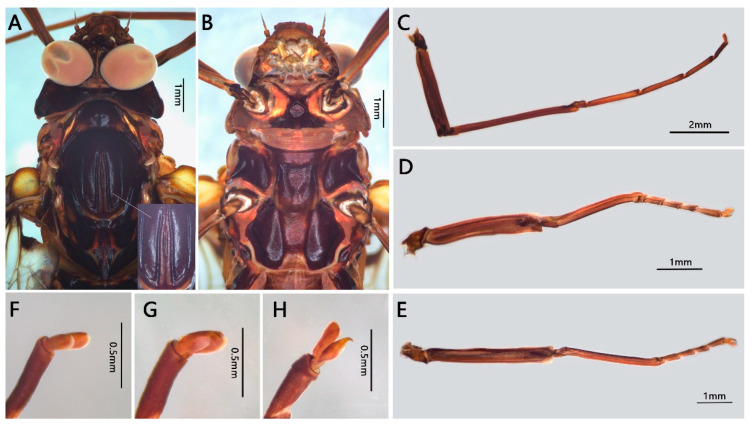
Male imago of *Pulchephemera projecta*: (**A**) head and thorax (dorsal view); (**B**) head and thorax (ventral view); (**C**) foreleg; (**D**) middle leg; (**E**) hindleg; (**F**) claw (foreleg); (**G**) claw (middle leg); (**H**) claw (hindleg).

**Figure 8:**
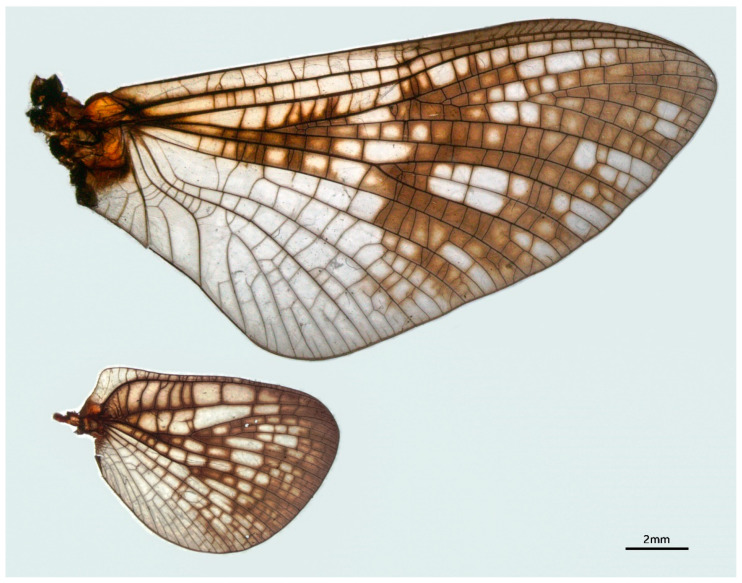
Wings of male imago of *Pulchephemera projecta*.

Male imago: body length greater than 16.0 mm, wings with large yellowish brown to reddish markings (Figure 6B); forelegs with two blunt claws, mid- and hindlegs with one sharp and one blunt claw (Figure 7F–H); basal C-Sc crossveins of forewings well-developed (Figure 8); both forewings and hindwings with relatively numerous crossveins; MA of forewing forked at middle point, MP forked at base; hindwings with round costal projection, MA unforked, MP and Rs forked near middle; forceps 4-segmented, basal segment half-length of second segment, segmentation of apical segments incomplete; penes fused with small median cleft, penis lobes with clearly thickened lateral margins; three caudal filaments subequal in length and well developed. 

**Figure 9:**
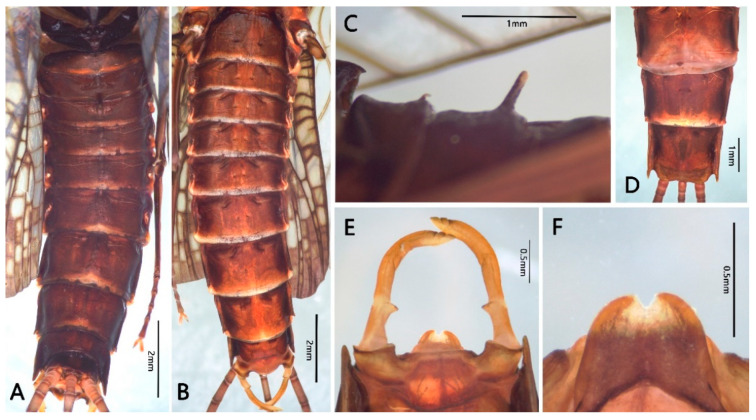
Abdominal structures of adults of *Pulchephemera projecta*: (**A**) abdominal terga of male imago; (**B**) abdominal sterna of male imago; (**C**) abdominal terga I–II (lateral view) of male; (**D**) sterna VII–IX of female imago; (**E**) genitalia (ventral view); (**F**) penis lobes (dorsal view).

Female imago: body greater than 16.0 mm in length, both forewings and hindwing with markings as in male; hindwings with rounded costal projection as in male; subgenital plate and anal plate with straight posterior margins (Figure 9D). 

**Figure 10:**
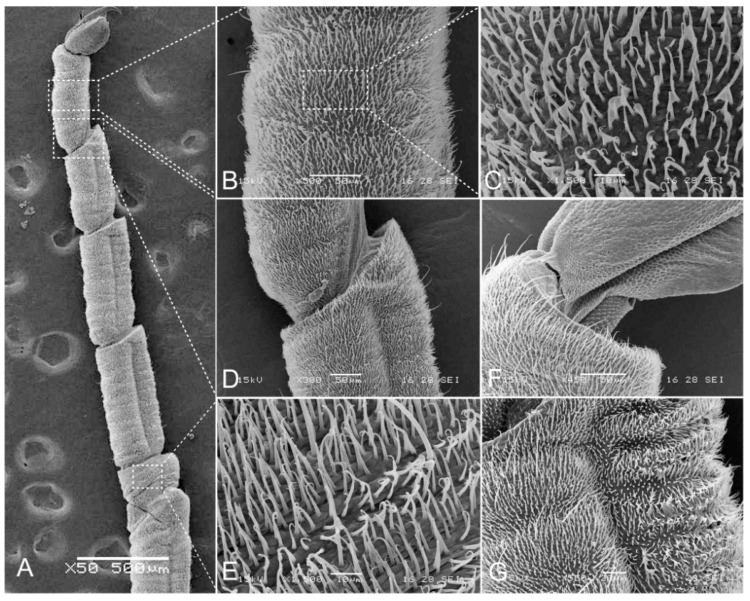
Subimaginal tarsi of *Pulchephemera projecta*: (**A**) tarsi of foreleg (male subimago); (**B**,**C**) tarsal segment 5 of foreleg and the details (male subimago) (**D**) base of tarsal segment 5 of foreleg (male subimago); (**E**) details of tarsal segment 1 of foreleg (male subimago); (**F**) apex of tarsal segment 5 of midleg (male subimago); (**G**) tarsal segment 4 of hindleg (female subimago).

Subimago: all tarsal segments of both male and female subimagoes only with microtrichia (Figure 10).

**Figure 11:**
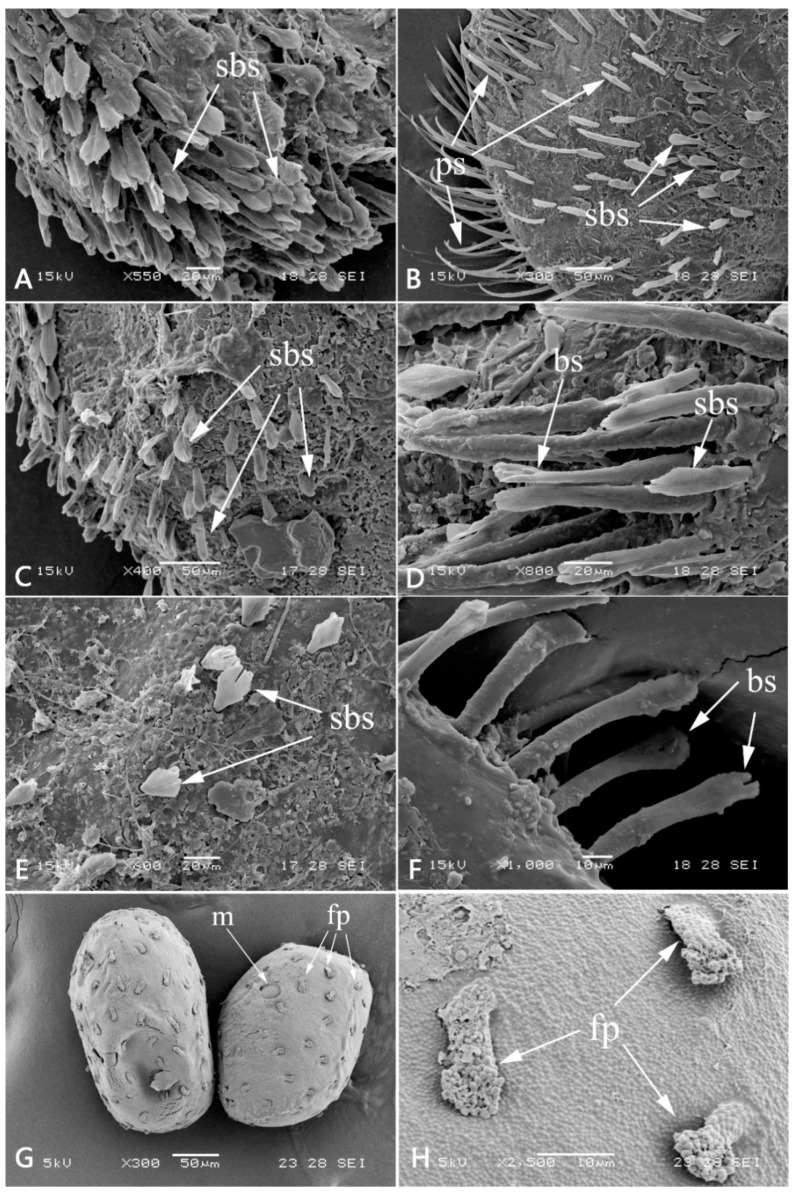
Setae of nymph and eggs of *Pulchephemera projecta*: (**A**) anterior margin of frontal projection; (**B**) dorsal view of labrum (left half); (**C**) lateral margin of mandible; (**D**) transverse row of setae on fore femora; (**E**) setae on diagonal ridge of gill II; (**F**) posterior margin of abdominal tergum VII; (**G**) eggs; (**H**) detail view of surface of eggs. Abbreviations: m, micropyle; sbs, stout bifurcate setae; ps, plumose setae; bs, bifurcate setae; fp, finger-like projections.

Egg: long oval, with finger-like projections on surface (Figure 11G). 

Diagnosis: (see Table 1) nymph with obvious frontal and genal projections, distinct anterolateral projections on pronotum, legs relatively short (tibiae shorter than femora), caudal filaments without swimming setae. Adults with colorful wings, rounded costal projection of hindwings, 4-segmented forceps and well developed median caudal filament.

Remarks. Compared to the Eastern Asian *Potamanthellus*, this new genus has several different characters both in nymphal and imaginal stages: in nymphal stage, *Pulchephemera* has no swimming setae on caudal filaments and the projections of pronotum and mesonotum are distinct; in imaginal stage, the genitalia and terminal filament are well-developed.

In contrast to the central Asian genus *Ochernova*, this new one possesses shorter legs (tibiae shorter than femora) and caudal filaments (ca 0.45–0.5× length of body) in nymphal stage. In imaginal stage, the coloration pattern of wings and the costal projection of hindwing of these two genera are totally different (see Table 1).

In the genus *Leucorhoenanthus*, the projections of pronotum and mesonotum are inconspicuous which are well-developed in our new genus. In addition, the adults of *Leucorhoenanthus* also have the colorless wings and vestigial terminal filament (see Table 1).

The American genus *Neoephemera* possesses unique characters in both nymphal and imaginal stages: tubercles always present on the nota of nymph (absent in all other genera including *Pulchephemera*) and the foreleg of male imago have one sharp and one blunt claw (which both blunt in *Pulchephemera*, *Leucorhoenanthu* and *Potamanthellus*).

This new genus has some combined characters of two groups in this family: the colorful wings and rounded costal projection of hindwings are similar to *Potamanthellus*; the 4-segmented forceps, fused penes and the absence of swimming setae in nymphal caudal filaments are alike to *Neoephemera*-like group. At the same time, the adults of this new genus have two unique characters and plesiomorphies at least: more crossveins on wings, MP and Rs of hindwings forked in the middle. The nymphs of the new genus have two characters of their own too: extended frons and genal projections. The large body of this new genus are also contributive to their identification. The forewing Cu area (between CuA and CuP) of the new genus is larger than that of *Neoephemera* but smaller than *Potamanthellus*. 

Eggs are slightly longer and with scattered finger-like projections on surface than those known eggs in the family. 

Etymology: the genus name *Pulchephemera* is derived from Latin “*pulch-*” and “*ephemera*” which means beauty or beautiful mayfly and refers to the colorful wings of the adults.

### 3.1. Description

*Pulchephemera projecta* (Zhou and Zheng, 2001) comb. n.

*Neoephemera projecta* Zhou and Zheng, 2001: 328, Figure 1, Figure 2, Figure 3, Figure 4, Figure 5, Figure 6, Figure 7, Figure 8 and Figure 9, nymph. Type: nymph, from Yunnan and Sichuan, China. 

*Neoephemera projecta*: Kluge, 2004: 276; Bauernfeind and Soldán, 2012: 514; Holland et al., 2016: 140.

Material examined. Mature nymph (Holotype), Chuan-Zhu-Shi (32.39° N, 103.35° E), Songpan County, Sichuan Province, 18-viii-2000, ZHOU Chang-fa. 3 nymphs (Paratypes); same data as holotype; 8 nymphs, Chuan-Zhu-Shi, Songpan County, Sichuan Province, 11-viii-2000, ZHOU Chang-fa; 12 male imagoes, 15 female imagoes, 2 male subimagoes, 1 female subimagoes, 25 nymphs, Potatso National Park, Shangri-La City, Yunnan Province, 1–25-iv-2021, MA Zhen-xing and MU Pen-xu; 5 nymphs, Qinggangshu Village, Xi’an City, Shaanxi Province, 7-viii-2019, HAN Na and ZHANG Ming. Material deposited in the Department of Biology, Nanjing Normal University, Nanjing, China.

Nymph: body length 19.0–22.0 mm (mature), caudal filaments length 8.0–10.0 (0.45–0.5× body), body yellowish brown (immature) to brownish black (mature) (Figure 1).

Head: generally brownish black. Antennae with dark base, other portion pale or colorless, slightly longer than head width, articulations with tiny setae; frons expanded and forming a distinct projection, anterior margin slightly to moderately concave (obviously concave in specimens of Shaanxi but nearly straight in specimens of Yunnan); Genae enlarged into clear projections (Figure 2A). Dorsal surface of head with scattered furcate-stout setae, margins of frontal projection and genal projections with more such setae (Figure 2A and Figure 11A); head vertex coarse (Figure 2A). Labrum: anterior margin slightly concave medially (Figure 2B), dorsal surface densely covered with setae which look like long plumose, stout plumose or bifurcate (Figure 11B); ventral surface densely covered with long and hair-like plumose setae. Mandibles: outer margin and dorsal surface densely covered with bifurcate setae (Figure 11C); outer incisor with 3–4 teeth, inner incisor with 2–3 teeth; prostheca divided into a tuft of fine setae and a single tooth; inner margin near mola with a row of dense fine setae (Figure 2C,D). Hypopharynx: anterior margin of lingua slightly concave medially, covered with dense fine setae; superlinguae with long hair-like setae apically (Figure 2E). Maxilla: galea-lacinia with 3 canines and 2 dentisetae, both inner and outer margins with row of long hair-like plumose setae; three segments of maxillary palp subequal in length, segment I distinct broader than segments II and III, with bifurcate-plumose setae along inner and outer margins, segment II with long hair-like setae, segment III with setae on distal half (Figure 2F). Labium: glossae with round pointed apex and subapical stout spine-like setae, ventral surface with dense long hair-like setae; paraglossae with dense long hair-like setae on both dorsal and ventral surface; segment I of labial palp expanded, segment II broader than segment III; length of segment I subequal to segment II, segment III ca. 2/3 length of segment II; segment I with dense bifurcate-plumose setae on ventral surface, segment II with stout bifurcate-plumose setae on ventral surface and long hair-like setae near outer margin; segment III with 7–10 subapical stout spine-like setae and covered with dense long hair-like setae near outer margin; mentum and submentum with bifurcate-plumose setae on ventral surface (Figure 2G).

Thorax: brown (immature) to brownish black (mature); pronotum expanded laterally, with distinct anterolateral projections; anterolateral projections of mesonotum extended to same level of pronotum (Figure 3A). Margins of thorax with dense stout bifurcate setae, dorsal and ventral surface of thorax covered with scattered stout bifurcate setae.

Legs: fore femora with a transverse row of bifurcate setae subapically (Figure 11D); femora of mid- and hindlegs similar, with scattered stout bifurcate setae on both dorsal and ventral surface; ventral surface of tibiae with scattered stout bifurcate setae; inner margins of both tibiae and tarsi with long plumose setae and fine setae. Claws of all legs with acute apex (Figure 3B–D). Length ratio of foreleg femur: tibia: tarsus = 1.9: 1.1: 1.5; midleg femur: tibia: tarsus = 2.1: 1.3: 1.3; hindleg femur: tibia: tarsus = 2.9: 1.7: 1.5. Patella-tibial suture of mid- and hindlegs clear expressed. Coxae and trochanter with setae too (Figure 3B–D).

Abdomen: brown to brownish black; terga I–II and VI–X with bifurcate setae along posterior margins and median line of each tergum (Figure 5A and Figure 11F); sterna covered with dense stout bifurcate setae. Terga I–II with distinct posteromedial tubercle (Figure 4A); segments V–IX with well-developed posterolateral projections, progressively larger posteriorly (Figure 5B). Gills I–VI present; gill I single, with 2 segments, apical one obviously longer than basal one and covered with long plumose setae and scattered fine setae (Figure 4D); gills II with dorsal subquadrate operculate plate meeting medially (Figure 4A,B), dorsal diagonal ridge on each operculum distinct and covered with scattered stout bifurcate setae (Figure 11E), free margins with long plumose setae and fine setae; ventral lamella much smaller than dorsal operculum, divided into several long fringes (Figure 4C). Gill III–V similar in shape and structure, dorsal lamellae “kidney-shaped” and with row of fringes on medial margin and lateral margin, ventral lamellae similar to gill II counterparts (Figure 4E–G); Gill VI single, similar to those dorsal lamellae of gills III–V (Figure 4H). Three caudal filaments subequal in length, with whorls of spine-like setae between articulations (Figure 5C).

Male imago: body length 17.0–20.0 mm, forewing 18.0–20.0 mm, hindwing 7.5–8.0 mm, caudal filaments 40.0–45.0 mm (2.3–2.5× length of body), body reddish brown to brownish black (Figure 6B).

Head: Compound eye: upper portion reddish grey, basal portion grey (eyes brown to dark in living) (Figure 6B and Figure 7A); distance between eyes subequal to width of median ocellus (about 0.10× dorsal diameter of one compound eye) (Figure 7A). Frontal projection with straight anterior margin. Vertex coarse (Figure 7A). 

Thorax: unicolorous reddish brown to dark brown, with a weak sutural ommation (Figure 7A); furcasternal protuberances dark brown, separated widely (Figure 7B). Forelegs darker than mid- and hindlegs; femora of all legs darker than tibiae or tarsi, with basal and apical dark dots (indistinct in mid- and hind femora) (Figure 7C–E). Ratios of fore femora: tibiae: tarsi = 2.9: 4.6: 5.4, ratios of fore tarsal segments I: II: III: IV: V = 0.2: 1.8: 1.5: 1.1: 0.7; Ratios of middle femora: tibiae: tarsi =2.5: 1.9: 1.8; ratios of hind femora: tibiae: tarsi = 3.5: 2.5: 1.9. First tarsal segment of mid- and hindlegs shorter than others, fifth segment longest, segments 2–4 subequal in length. Foreleg with two blunt claws; mid- and hindlegs with one acute and one blunt claw (Figure 7F–H).

Wings: almost entire forewings and hindwings covered with purplish to brown markings except Cu to A fields (Figure 8); both forewings and hindwings with relatively dense crossveins including basal portion of C and Sc. Forewings: Rs forked at midpoint between base of MA+Rs and fork of MA; MP forked near base; MP2, CuA and CuP recurved; CuP ends near midpoint of hind margin; A1 single, with two veinlets; bullae of Sc, Rs clear, crossveins of stigma oblique, some of them S-shaped. Hindwings ca. 0.4× forewing length, with rounded costal projection at base; MA single, Rs fork at about same level as MP fork (Figure 8).

Abdomen: reddish brown to dark brown (Figure 9A,B), terga I–II with posteromedial tubercles (reduced tubercles of nymph), that of tergum II much larger (Figure 9C); terga VIII–IX with clear posterolateral projections (Figure 9B), those of terga IX much longer; each sternum with a pair of short dark stripes and submedian paired dots, area near lateral margin darker than medial area (Figure 9B). Genitalia (Figure 9E,F): posterior margin of styliger plate straight; forceps yellow, paler than terga or subgenital plate; segment I of forceps broader than others, with clear inner projection apically; segment II ca. 2.0× length of segment I, curved inwards; segments III and IV very short, segmented incompletely and tapered. Penes fused with median notch; penes very thin, sperm ducts along lateral margins and that area thicker than others. Three brown caudal filaments with tiny setae on surface, terminal filament slightly shorter than cerci (Figure 6B).

Female imago: body length 18.0–20.0 mm, forewing 20.0–22.0 mm, hindwing 8.0–8.5 mm, caudal filament length 30.0–34.0 mm (1.5–1.7× length of body), coloration pattern similar to male imago (Figure 6D). Wings similar to male. All legs with similar coloration as male imago, length ratios of femora: tibiae: tarsi of forelegs = 3.0: 2.5: 2.6; length ratios of femora: tibiae: tarsi of middle legs = 2.8: 2.3: 2.2; length ratios of femora: tibiae: tarsi of hindlegs = 3.9: 2.9: 2.2; all legs with one blunt and one sharp claw. Abdominal coloration similar to male, subgenital plate slightly expanded posteriorly, posterior margin of subanal plate nearly straight (Figure 9D).

Male subimago: body length 19.0 mm, forewing 19.0 mm, hindwing 8.0 mm, caudal filaments 20 mm (ca 1.0× length of body); coloration similar to male imago but somewhat fainter (Figure 6A). Wings semi-hyaline and with tiny setae along margins. Legs darker than those of male imago, length of tarsi in forelegs subequal to tibiae, all legs with one blunt and one sharp claws; all tarsal segments of all legs covered by dense microtrichiae (Figure 10A–F). Caudal filaments with dense setae on surface, terminal filament slightly shorter than cerci.

Female subimago: body length 20.0 mm, forewing 20.0 mm, hindwing 8.5 mm, caudal filament length 20 mm (ca 1.0× length of body), coloration similar to male subimago (Figure 6C). All legs with one blunt and one sharp claws; same as male subimago, all tarsal segments covered by dense microtrichiae (Figure 10G).

Eggs: length 210–240 μm; width 140–150 μm, oval (Figure 11G), finger-like projections (fp) 12–16 μm, scattered on the surface (Figure 11G,H), these projections consist of many thin threads and somewhat expanded apically (Figure 11H). Tagenoform micropyle located in the equatorial area, sperm guide round, diameter 17–20 μm (Figure 11G). 

### 3.2. Biology and Remarks

**Figure 12:**
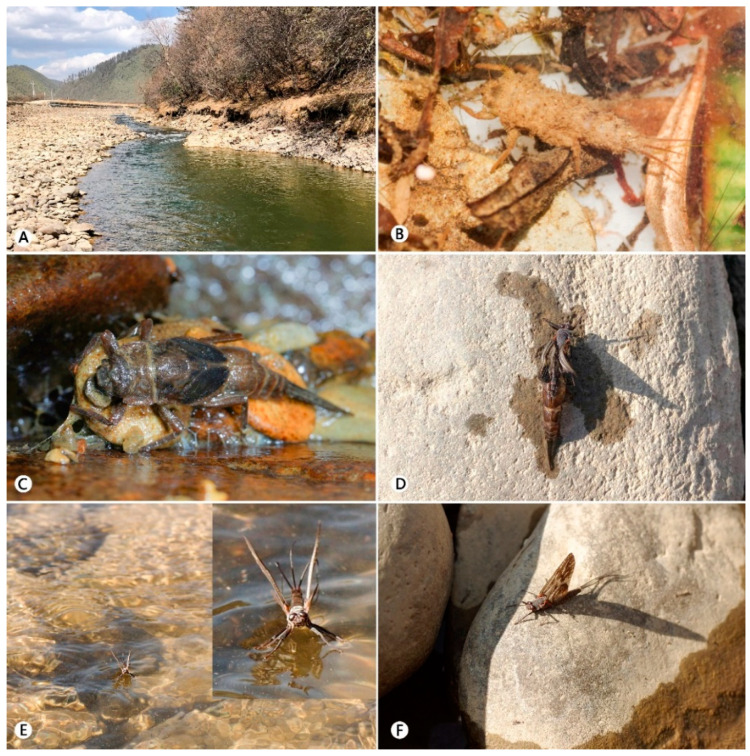
Habitat and emergence process of *Pulchephemera projecta*: (**A**) habitat; (**B**) immature nymph alive; (**C**) mature nymph alive; (**D**) subimago in the process of emergence; (**E**) subimago on the water surface; (**F**) subimago on the bank of the river.

Nymphs of this species (collected in 2021) live in small to middle sized streams (1.0–8.0 m wide, 0.1–1.5 m deep, ca. 2500–3400 m altitude, Figure 12A), and they are found underneath stones in moderate to fast flowing sections where the substrate is stony, most stone covered with mud and submerged plants (Figure 12C). The nymphs move slowly in the water, have weak swimming ability, their body are usually covered with dense muddy debris (especial immature nymphs, as Figure 12B).

Observed emergence happened from early to the end of April. Subimagoes emerged between 4:30 and 6:00 p.m. local time, and this process occurred at the surface of stream water (nymphs float to the surface of the water and then become subimagoes, see Figure 12D, the molting nymph in the photo was not in its natural state but was placed on a stone). After that, the subimagoes drift downstream with the water current on its surface (Figure 12E) and then climb onto stones or stream banks when they meet them (Figure 12F). 

Life span of subimagoes in the laboratory ranges from 53–68 h. Molting of the subimago to imago was observed both in evening and daytime, the process lasts ca. 14–17 min.

Distribution. China (Shaanxi, Yunnan and Sichuan).

Egg diagnosis. The eggs of this species are more similar to the species of the *Neoephemera*-group because of their finger-like projections [1]. Compared to eggs of *Neoephemera youngi*, *N. eatoni* and *L. maximus* [3,21], the finger-like projections of *Pulchephemera projecta* are stouter and irregularly situated. 

## 4. Discussion

Considering a series of plesiomorphies of *P. projecta* (such as large body and wing size, more crossveins, well-developed genitalia and terminal filament), we regard it as the most primitive taxon in Neoephemeridae. Therefore, the characters of its wings (with dark markings and hindwings with blunt costal projection) are also considered as the primitive state of this family rather than the synapomorphies of the new genus and *Potamanthellus*. For the same reason, the frontal projection in nymphal stage is also regarded as a plesiomorphy of this species and the family as well.

**Figure 13:**
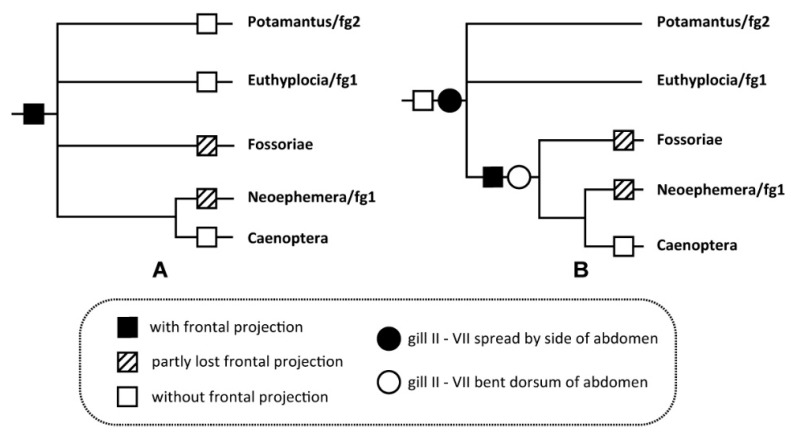
The two hypotheses of phylogeny of Fimbriatotergaliae: (**A**) hypothesis 1: frontal projection of nymph is the synapomorphy of Fimbriatotergaliae; (**B**) hypothesis 2: frontal projection of nymph is the synapomorphy of Caenotergaliae+Fossoriae.

Historically, the phylogenetic position of the family Neoephemeridae has been changed several times [1,5,10,11,22,23,24,25,26]. In the system of Kluge, the relationship between Potamantus/fg2, Euthyplocia/fg1, Fossoriae and Caenotergaliae (=Neoephemera/fg1+ Caenoptera) is unclear. Based on the characters of *P. projecta* described in this work, we propose two hypotheses on this issue (Figure 13). The first one is that the nymphal frontal projection is the synapomorphy of Fimbriatotergaliae, which then were secondarily lost in Potamantus/fg2, Euthyplocia/fg1 and Caenoptera. The second one hypothesizes that the frontal projection is the synapomorphy of Caenotergaliae+Fossoriae only. Meanwhile, the dorsal-oriented gills may also be another synapomorphy of Caenotergaliae+Fossoriae. Similar frontal projection found in some mayfly taxa which are not belonging to Fimbriatotergaliae, such as Drunella/g1 [5], is obvious homoplasy and the imaginal characters of them were totally different.

**Figure 14:**
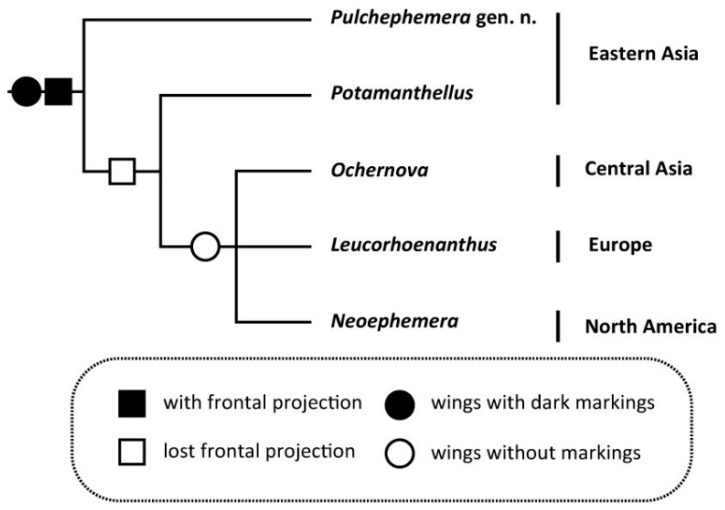
The hypothesis of the phylogenetic relation between the genera within Neoephemeridae (partly based on Bae and McCafferty [1]).

On the generic level, based upon phylogeny provided by Bae and McCafferty [1], we suggest a new hypothesis for our new genus (Figure 14). In this arrangement, the genus *Pulchephemera* was regarded as the first divergent taxon of Neoephemeridae. The rest genera are grouped by the absence of frontal projection. Among them, the *Neoephemera*-like group left the *Potamanthellus* because they lost paintings on wings and have acute costal projection on hindwings. 

Geographically, we believe that the family Neoephemeridae originated from Eastern Asia since our new primitive genus is in this region, then dispersed western-wards. During this process, the other genera split and originated progressively. The most plesiomorphic mayfly *Siphluriscus chinensis* Ulmer, 1920 is distributed in the Orient too [27], and this region also has the most diverse species of the families Ephemeridae and Potamanthidae. So far, several studies of genus or family-level phylogeny for mayfly based on molecular data have been published, and they either verified the traditional morphological results or proposed new hypotheses [28,29,30]. Therefore, analyses for phylogenies of Neoephemeridae based on molecular evidence are necessary to test our theory in future work. 

## Figures and Tables

**Table 1:** Comparison of characteristics of five genera within Neoephemeridae.

	Genera	*Neoephemera* [1,3,5,18]	*Ochernova* [1,5,9]	*Leucorhoenanthus* [1,5,6,19,20]	*Pulchephemera* gen. n.	*Potamanthellus* [1,5]
Characters						
Nymph	Frontal projection on head	Absent	Absent	Absent	Present	Absent
	Mesonotal anterolateral expansions	Greatly or somewhat expanded	Somewhat expanded	Without distinct expansions	Somewhat expanded	Without or somewhat expanded
	Maxillary palpi (length ratio of terminal segment/2nd segment)	0.7–1.3	0.35	0.78–0.84	1.0–1.1	1.1–2.0
	Labial palpi (length ratio of terminal segment/2nd segment)	0.6–1.1	0.39	0.65	0.65	1.0–1.5
	Patella-tibial suture of mid- and hindlegs	Present	Absent	Present	Present	Present
	Length of mid- and hind femora	Longer than tibiae	Shorter than tibiae	Longer than tibiae	Longer than tibiae	Longer than tibiae
	Median tubercles on thoracic terga	Well developed (except *N. eatoni*)	Absent	Absent	Absent	Absent
	Caudal filaments	Without swimming setae; ca. 0.8–1.0× length of body	Without swimming setae; ca. 1.4× length of body	Without swimming setae; ca. 0.7× length of body	Without swimming setae; ca. 0.45–0.5× length of body	With swimming setae; ca. 0.4–0.7× length of body
Male imago	Ratio of distance between compound eyes/diameter of compound eyes	0.15–0.5	/	1.15	0.1	0.04–0.15
	Claws of forelegs	One sharp, one blunt	/	Two blunt	Two blunt	Two blunt
	Forewing length (mm)	8–17	9	8–11	18–20	6–10
	Coloration of wings	Without markings	Without markings	Without markings	with distinct markings	with distinct markings
	Basal C-Sc crossveins of forewings	Reduced	Reduced	Reduced	Not reduced	Not reduced
	Shape of basal costal projection of hindwings	Acute	Acute	Acute	Rounded	Rounded
	Forceps	Well developed, 4-segmented	Well developed, 4-segmented	Well developed, 4-segmented	Well developed, 4-segmented	Vestigial, 3-segmented
	Median incision of penis	Small	Small	Small	Small	Wide
	Median caudal filament	Well developed	Well developed	Vestigial	Well developed	Vestigial
	Length ratio of cerci/body	1.0–1.5	/	2.4	2.3–2.5	2.2–4.5

## Data Availability

All data is available in this paper.
